# Domestic violence routine screening in a public dental hospital

**DOI:** 10.1038/s41405-025-00386-w

**Published:** 2026-01-14

**Authors:** Cecilia Correy, Natalia Uthurralt, Carolyn Day, Renee C. Lovell, Alice Kucera, Shilpi Ajwani

**Affiliations:** 1 Faculty of Medicine and Health, The University of Sydney, NSW Sydney, Australia; 2 Sydney Dental Hospital, Sydney Local Health District, Sydney, NSW Australia; 3 NSW Ministry of Health Centre for Oral Health Strategy, NSW Sydney, Australia; 4 The Edith Collins Centre (Translational Research in Alcohol, Drugs and Toxicology), Sydney Local Health District, Australia Community Health, Sydney Local Health District, Sydney, NSW Australia; 5 Community Health, Sydney Local Health District, Sydney, NSW Australia

**Keywords:** Dental trauma, Dental public health

## Abstract

**Introduction:**

Intimate partner violence (IPV) is a major public health issue and leading cause of early death for women in Australia and internationally. Implementation of Domestic Violence Routine Screening (DVRS) in public health settings has been found as an effective way to identify IPV and offer support.

**Aims and setting:**

To evaluate DVRS implementation in adult and paediatric clinics of a public dental hospital in Sydney, Australia, from January 2023 until February 2024.

**Design:**

Prospective clinical study where eligible women were screened for IPV using a tool adapted from standardised screening tools for use across multiple public health services.

**Materials and methods:**

Demographic, medical, dental, psycho-social data and emergency department presentations were analysed from health records.

**Results:**

The study clinics cared for 10,197 women and 5597 carers over 13-months. In the 6-months post implementation, IPV disclosure increased by 529% (7–44 cases). By month 13, the IPV disclosure rate was 11% (*n* = 85). Screening did not impede the dental appointment. All women who disclosed IPV were offered referral to support and 36 women (42%) accepted.

**Conclusion:**

DVRS implementation resulted in an IPV disclosure rate comparable to other public health services where DVRS is mandatory. Public dental services can play an important role in screening and supporting women who have or are experiencing IPV.

## Introduction

Domestic violence (DV) is considered a major public health issue and leading cause of early death for women in Australia and internationally [1–6]. DV includes any behaviour, in an intimate or family relationship, which is violent, threatening, coercive or controlling, causing a person to live in fear [2, 7]. Intimate partner violence (IPV), is a specific form of DV inflicted by a person’s current or former intimate partner and may include physical, sexual, and emotional abuse [4]. Globally, women are overrepresented as victims of IPV, with one-in-three women experiencing some form of IPV in their lifetime [3]. In Australia, data indicates that one-in-four women experience IPV [8], with First Nations women subjected to IPV at rates 2–5 times higher than other Australian women [6], reflecting the ongoing impacts of colonisation, including intergenerational trauma [1, 6, 9, 10].

IPV has detrimental impacts on a women’s physical and mental health [1–4, 11]. Women subjected to IPV are more likely to utilise mental health, drug health, Emergency Department (ED) and outpatient health services. They are more likely to develop diabetes, chronic health and pain conditions, sexually transmitted infections and have worse symptoms of menopause[2, 12–14]. Globally, approximately one-in-three woman experience IPV by a current or previous partner within their lifetime and the World Bank Group estimates that global Gross Domestic Product (GDP) costs of IPV are between 1.2 and 2% [15]. In Australia in 2024, on average, one woman was killed every four days by a current or former intimate partner and the estimated cost in 2021–22 was $15.6 Billion [7, 16].

IPV is rarely disclosed to service providers without specific prompting and is therefore at risk of going undetected [17]. DVRS is a structured practice whereby all patients are screened for IPV, when safe to do so, compared to targeted screening which focuses only those presenting with risk factors or specific and specified vulnerabilities, such as a visible injury [18]. Safe screening involves asking the questions face-to-face when the woman is alone with the clinician, away from potential perpetrators of violence, so the questions can be asked without putting them at further risk. Health services can offer safe, trauma-informed support for women subjected to IPV [8, 19]. However, if health service providers fail to respond to IPV disclosure appropriately, it can be disempowering and retraumatising for women [18, 20]. Implementing domestic violence routine screening (DVRS) in health services has been found to increase detection of IPV and facilitate a person-centred, trauma informed and evidence-based response [17]. For this to occur, staff education, privacy, prompt response and referral pathways are required [17, 21].

Healthcare IPV screening practices vary across health systems globally, with screening taking place in settings like obstetrics, community health and EDs in North America, United Kingdom, and parts of Asia [19, 22]. Similarly to Australia, screening practices vary between routine or targeted and are conducted by various health professionals, including nurses, midwives and social workers [19, 22, 23]. Some Australian states mandate DVRS in public health services where women at high-risk present [24]. In the state of New South Wales (NSW), DVRS has been mandatory in public mental health, drug health, early childhood, and antenatal services since 2003. Screening in mandated in drug health and mental health because a large percentage of the client group is known to have a number of complex interlinked presentations or challenges. These challenges include substance use disorders, mental health issues, multiple ED presentations, involvement with child protection services, homelessness and/or financial stressors [25]. Screening is mandated in antenatal and child and family health services because, despite low IPV disclosure rates, pregnancy and the early childhood period are high risk time periods for IPV [18, 25]. Screening questions focus on whether a woman has been subjected to IPV by a current or former partner within the last 12 months. This timeframe includes former partners because after a relationship ends is known to be a high-risk period for perpetrators inflicting IPV.

DVRS studies report that women generally feel it is appropriate for health workers to inquire about IPV. Additionally, healthcare staff often recognize DVRS as an integral part of their role, understanding its importance in providing comprehensive care [21, 24, 26, 27]. Implementing DVRS in healthcare settings can lead to better identification of IPV cases, enabling timely counselling, referrals, and interventions to improve health outcomes [24].

Dental settings and particularly, public oral health settings are another potential location for DVRS. Similar to the drug health and mental health settings, a significant proportion of the population attending public oral health clinics are likely to be experiencing multiple layers of disadvantage and are at higher risk of chronic mental health conditions, substance use disorders and disabilities [13]. These issues are known to increase risk and severity of IPV [21, 28]. Exposure to IPV has also been linked to higher rates of caries and poorer oral health in children [29]. Indeed, there is a growing international awareness of a need for oral health services to develop the capacity of dental clinicians to identify and respond to IPV [30–33]. The limited research relating to dentists’ knowledge of IPV and perspectives on their role [10, 34] has found that patients may disclose IPV in their appointments or present with injuries, and that dentists feel that responding to IPV is part of their role [10, 31, 34].

Despite the increasing call to action, there is limited targeted interventions to IPV in dental settings [25, 35] and to our knowledge there are no publicly funded dental clinics that routinely screen for IPV in Australia or internationally. We report on the results of a pilot study, one of the first of its kind, of DVRS in a public dental setting [26, 30, 31]. Specifically, the primary aim of this study was to determine whether DVRS, when delivered in a public dental setting, increases IPV identification. The secondary aims were to:

I. gain an understanding of characteristics present in public dental patients who disclosed IPV, along with their support and referral needs.
II. assess if DVRS impeded women’s (or their children’s) planned dental treatment.

## Methods

### Study setting and design

This pilot study was set within a publicly funded Dental Hospital in metropolitan Sydney, Australia. Sydney Dental Hospital (SDH) is a tertiary dental facility with 143 public dental chairs that is open 08:00-16:30 weekdays. The service provides early intervention, oral health promotion and community treatment services to people with complex health and social needs. SDH was selected as an ideal site as it sees a high proportion of patients from culturally and racially diverse groups, who experience high levels of disadvantage. Additionally, due its location, there is higher proportion of patients who are homeless, and from high density public housing and boarding houses and known to be at higher risk of IPV.

DVRS was introduced in January 2023 for all eligible female patients or carers aged 16-years or over, in a paediatric dentistry clinic and one adult oral health clinic at SDH.

### Implementation

#### Protocol development

The procedure outlined how to access and use the NSW Health DVRS tool, information about safety, processes for documentation, referral pathways and district health service policy on when escalation is required [25, 27]. The screening tool was developed by NSW Health, adapted from standardised screening tools for use across multiple public health services [36]. It also includes how staff respond to disclosures of IPV, including immediate safety planning in cases where women and children were currently at high risk, including processes to transfer care to local adult and children’s emergency departments within the same health district, if further support out of hours was needed.

#### Training

All staff in the participating clinics received a full day of IPV and DVRS education provided by an accredited trainer. It included information on health impacts of IPV, how to explain and conduct the DVRS, understanding referral and support options, how to handle disclosures of IPV, including risk assessment, safety planning and mandatory reporting requirements related to the safety of women and children [25, 27]. Staff received further training tailored to their clinic setting by the senior social worker who provided information on the safe screening protocols for each clinic area. All clinical staff are also required to undertake Child Protection training as part of employment and are aware of child protection protocols if risk issues are identified.

#### Electronic form

The NSW Health DVRS tool was developed into an electronic form and embedded in the Oral Health electronic record, Titanium [25, 27]. It included the following questions (i) within the last year have you been hit, slapped or hurt in other ways by your partner or ex-partner; (ii) are you frightened of your partner or ex-partner. If question 1 or 2 were answered “yes”, women were then asked questions about any immediate danger and safety of themselves and/or their children [28]. Women who disclosed IPV were offered social work, legal support and a discrete IPV pamphlet [17, 27].

#### Eligibility

All female patients, parents, or carers aged 16-years and over were eligible to be screened when it was safe to do so, specifically when women were alone. In Paediatric Dentistry the protocol was adapted to the local workflow and screening was undertaken when the children were taken out of the clinic room by the Dental Assistants to have their height and weight measured or for radiographs. If IPV is disclosed, the Dental Assistants could allow the clinician further time alone with the woman in order to assess immediate safety and offer support.

#### Data collection and analysis

SDH records were reviewed to determine the prevalence of IPV disclosure to identify (i) prevalence of disclose pre- and post-interventions; (ii) characteristics of those women who disclosed.

Data extracted from the electronic medical records of women who disclosed IPV in the 6-months prior- and 13-months post-DVRS implementation included demographic (age, gender, Australia born, preferred language and postcode), medical, dental, psycho-social data and ED presentations. For women who disclosed IPV, referrals to social work, presentations to the ED, demographic and health data were also extracted. Pre-intervention IPV cases were identified by data mining electronic medical records of all referrals to social work in the 6 months prior to DVRS implementation and reviewing the reason for referral. Information was not available on whether the women had been asked about IPV directly by their clinician or voluntarily disclosed IPV. Post-implementation cases were extracted from the imbedded DVRS form in Titanium.

#### Statistical analysis

Screening and response data were deidentified and analysed using R version 4.2.3. Quantitative data were analysed using descriptive statistics. Frequencies with percentages and means with standard deviations were used to describe the sample distribution. Medians and corresponding ranges were used where data was not normally distributed.

#### Ethical considerations

The procedure for screening at SDH was developed in conjunction with the NSW Health department’s Prevention and Response to Violence Abuse and Neglect unit (PARVAN) and the SLHD Domestic Violence Strategy Unit. This process outlines that women can only be screened when they are alone and the clinician follows all measures to protect their safety.

The study was approved by the Royal Prince Alfred Hospital, Human Research Ethics Committee (HREC; X22-0298 & 2022/ETH01761). In compliance with the National Statement on Ethical Conduct in Human Research 2007 [37], and to ensure victim-survivors’ safety and limiting unintended consequences, a waiver of consent was sort and received for pre-DVRS and post-DVRS implementation clinical data. All data was extracted from medical records on site by the research team and entered into a database hosted by SLHD. All patient identifiers were removed at the point of data extraction ensuring patient privacy and confidentiality.

## Results

**Fig. 1:**
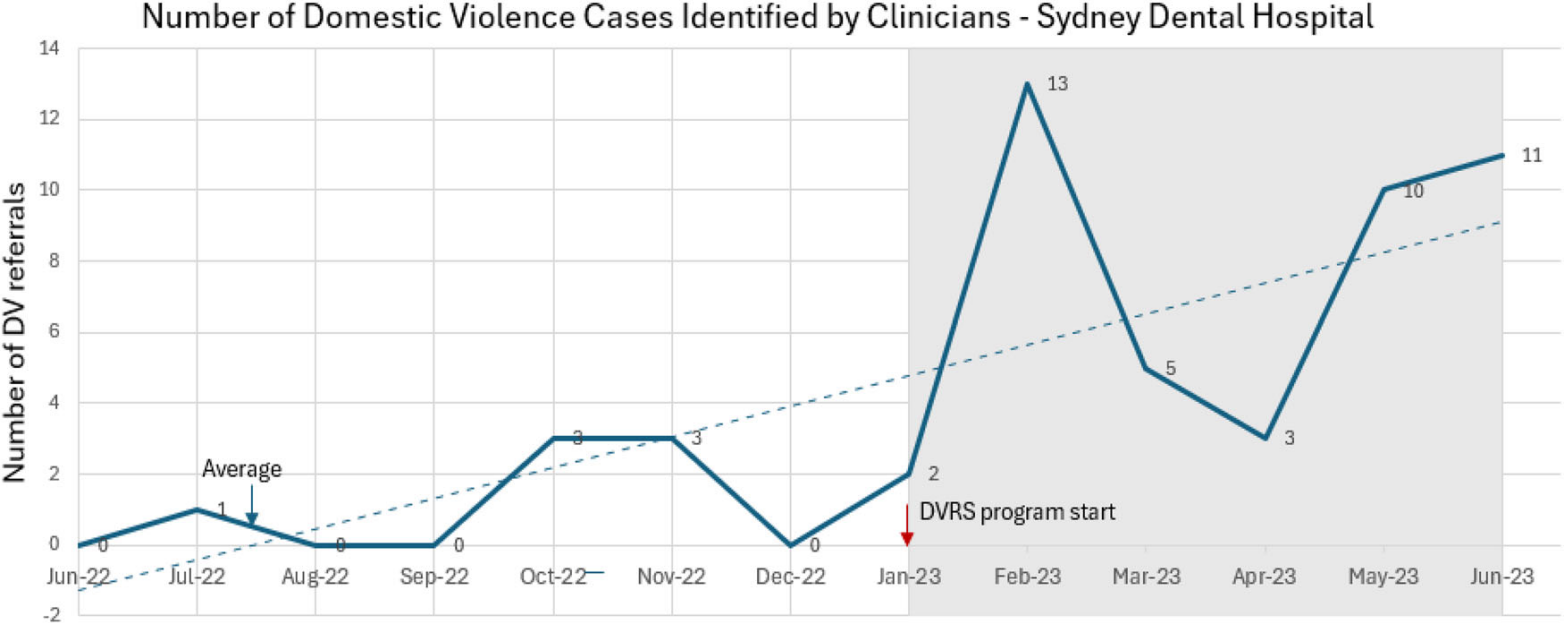
Number of IPV cases identified by pre and post DVRS implementation.

In the 6 months prior to the implementation of the DVRS (June to December 2022), clinicians identified only seven IPV cases. However, during the first 6 months of the DVRS pilot, clinicians identified 44 cases of women who had experienced IPV by a current or former partner in the last 12 months, representing a significant 529% increase in identified cases from pre- to post-DVRS implementation over the same period (Fig. 1).

**Fig. 2:**
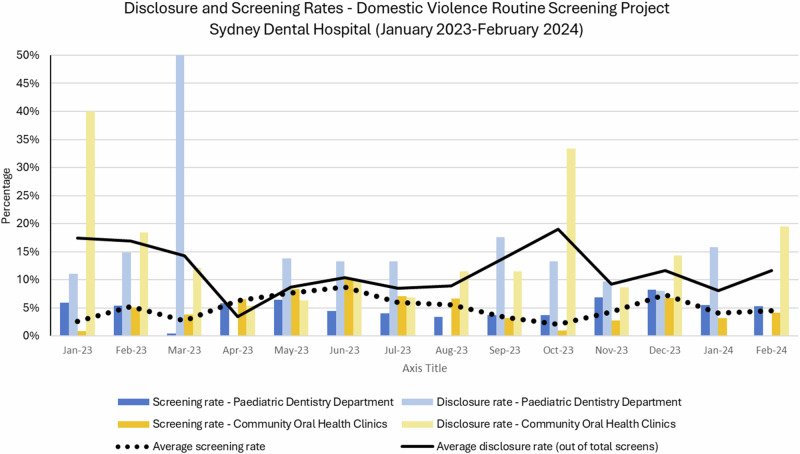
Disclosure and Screening Rates – January 2023 – February 2024.

During the 13-month study period (January 2023 to February 2024), 10,127 women attended the Community Oral Health Clinic (COHC), and 5597 carers/parents attended the Paediatric Dentistry at the Sydney Dental Hospital, with 789 women were screened for IPV in total (Table 1). The screening rate in the COHC was calculated using the formula: ${{\rm{Screening}}}\; {{\rm{rate}}}=\frac{{{\rm{Number}}}\; {{\rm{of}}}\; {{\rm{women}}}\; {{\rm{screened}}}}{{{\rm{Total}}}\; {{\rm{number}}}\; {{\rm{of}}}\; {{\rm{eligible}}}\; {{\rm{women}}}}{{\rm{x}}}100$. However, in the paediatric clinic, it was impossible to calculate the screening rate accurately because the gender of parents/carers is not identified. Therefore, the denominator for this clinic was the total number of appointments, resulting in a lower overall screening rate. The overall screening rate ranged between 4% and 9%, with an average of 5% over the study period (Fig. 2).

**Table 1:** Disclosure and Screening Rates, January 2023 – February 2024.

	Females presenting to Community Oral Health Clinics	Carer/ parent presenting to Paediatric Dentistry	Total
Number of women eligible to be screened	10,127	5597^a^	15,724
Number of forms completed	731	966	1788
Number of women screened	516	273	789
Screening rate^b^	5%	5%	5%
Number of clients IPV identified	57	28	85
Disclosure rate^c^	11%	10%	11%

^a^Data could not be separated into patients eligible accompanied female carers and appointments; the figure represents all appointments in Paediatric Dentistry in the study period.

^b^Screening rate = Number of women screened/Total number of eligible women.

^c^Disclosure rate = Number of women who disclosed DV/Total number of women screened.

The disclosure rate was calculated using the formula: ${{\rm{Disclosure}}}\; {{\rm{rate}}}=\frac{{{\rm{Number}}}\; {{\rm{of}}}\; {{\rm{women}}}\; {{\rm{who}}}\; {{\rm{disclosed}}}\; {{\rm{IPV}}}}{{{\rm{Total}}}\; {{\rm{number}}}\; {{\rm{of}}}\; {{\rm{women}}}\; {{\rm{screened}}}}{{\rm{x}}}100$. Out of 789 women screened, 85 reported experiencing intimate partner violence (IPV), resulting in an overall disclosure rate of 11%. The disclosure rate peaked at 19% and reached a low of 3%, with the lowest rate observed during April 2023 (Table 1 and Fig. 2).

### Demographics and risk factors

Of the 85 women who disclosed a history of IPV in the past 12-months, 29 (34%) reported being hit, slapped or hurt in other ways by their partner or ex-partner and 23 women (27%) reported being frightened of their partner or ex-partner. There were 31 women (39%) who reported an occurrence of physical IPV in the past 12-months and being frightened of their partner or ex-partner.

The mean age of women who disclosed IPV was 44-years. Country of birth was available for 83 of the 85 women (98%) with 55 (65%) born in Australia. Similarly, preferred language was documented for 83 women with 74 (89%) reporting their preferred language as English while 9 (11%) preferred another language. Nearly one-third of the women (31%, *n* = 26) were public housing residents, but housing information was unascertainable for 28 (33%) women. Almost 50% of women (*n* = 40) reported insecure or no employment, but employment data was unavailable for over one-third of cases (35%, *n* = 30). More than three-quarters of the women were found to have social and support needs (*n* = 65) and 17 (20%) women had personal involvement with the justice system. Medical records indicated that for 27 women (32%), the perpetrator of IPV had known involvement with the criminal justice system. This information was not stated in the medical record for 52 of cases (61%).

Of the women who disclosed IPV, 56 had children (66%), and 22 (39%) had current involvement with child protective services, and four (7%) had past involvement. Four women (5%) had personal experience of being in out-of-home (foster) care, and 11 women (13%) currently had children in out-of-home care.

Women who disclosed IPV also often reported a cooccurrence of social support needs (such as housing and legal difficulties, community and family support services) parenting difficulties; a mental health diagnosis; and a substance use disorder; a diagnosis of a disability; and insecure/inadequate housing (Table 2).

**Table 2:** Prevalent mental, behavioural, and neurodevelopmental health conditions.

	*N*	%
Depression	26	50
Anxiety	16	30.8
Post-traumatic stress disorder	15	28.8
Polysubstance abuse	14	26.9
Suicidal ideation	10	19.2

Of the women who disclosed IPV, 78% were found to have at least one medical diagnosis in their records. Mental, behavioural, or neurodevelopmental conditions accounted for 61% of the reported conditions (*n* = 52; Table 2). Depression was the most prevalent condition and reported in 50% of cases. Other commonly occurring conditions were anxiety (31%), post-traumatic stress disorder (29%), polysubstance use (27%). Other conditions included suicidal ideation and self-harm, mental health diagnoses including borderline personality, bipolar and schizoaffective disorder, postnatal depression, schizophrenia and other substance use disorders. Twenty-four women (28% of IPV disclosures) reported being diagnosed with a disability.

### Emergency presentations

Medical ED presentations were analysed over the preceding 12-months prior to the SDH appointment where IPV was disclosed. Of the 85 women who disclosed IPV, data were available for 83. Over that 12-month period, 33 women (39%) had at least one ED presentation, with nine women (11%) presenting more than three times (Table 3). Many presentations involved multiple reasons, with mental illness (39%, 13 cases) and trauma (defined as a physical injury in 9 women) being the most common. Specific data on the type of physical trauma was not collected.

**Table 3:** Individual ED presentations.

	*N*^a^	%
Number of presentations where DV was identified at the ED	6	6%
Number of presentations where DV was not identified in medical record	58	70%
Presentations with insufficient data to determine DV identification	19	24%
Total recorded individual ED presentations	83	

^a^Different cases.

All 85 women who reported IPV were referred to the SDH senior social worker and/or legal support service. In total, 36 women (42%) accepted the referral from dental clinicians. Of the 36 women who accepted support, 97% received SDH social worker support and 47% received legal aid. Eleven women (31%) were referred to the government victim support scheme which assists victims of violent crime, modern slavery and road crime in NSW and eight (22%) received housing support. Seventeen women (47%) also received counselling services.

### Impact on dental treatment

Screening for IPV did not impede the completion of dental treatment required on the day of screening for patients in both the adult and paediatric clinics, with 789 women completing their treatment after being screened. All 85 women who disclosed IPV completed their dental appointment. Out of the 85 women, 77% had a scheduled follow-up appointment. Among those with scheduled follow-up appointments, the majority (96%) attended their appointments. The women who did not attend their follow-up appointments had a history of non-attendance, with more than three missed appointments in the previous 12 months. Data was not available on why the 4% of women did not attend their follow up appointment.

## Discussion

To our knowledge, this is the first study of DVRS conducted in a public oral health setting. The findings contribute to the growing body of evidence that IPV interventions in oral health settings are not only effective, but essential. This study found that it is feasible to screen for IPV in a public oral health setting and that DVRS in a highly effective way to improve detection of IPV, with more than a 500% increase in IPV identification following DVRS implementation and concomitant increase in access to other forms of support for women with complex needs. Importantly, DVRS did not appear to impede the patient’s treatment on the day of screening or their attendance of future appointments. The prevalence of IPV identification in our sample was 11%, a prevalence similar to that mental health and drug health services in the Health District, where DVRS is mandated and many clients typically have complex needs. However, the prevalence is much higher than antenatal and child and family health, where DVRS is also mandated. This justifies the exploration of mandatory DVRS being implemented in the public dental space.

Recent Australian studies have found that both adult and child public dental patients experience higher proportions of social disadvantage [38, 39]. Our data supported these findings as the majority of women who disclosed IPV had multiple risk factors/complex needs, a similar proportion to those attending public mental health and drug and alcohol services. Intersectionality and factors such as substance use disorders, chronic mental health conditions, homelessness and disability are associated with higher morbidly and mortality, and experience difficulties accessing support in relation to IPV [2, 8, 12]. The identification of risk factors in this study highlights the opportunities for increasing care and support for patients in the public oral health system. Literature on women subjected to IPV highlights the importance of documenting acts of resistance, strength and resilience [40]. These were present in that the EMR notes of the women who disclosed IPV however, this data was not collected, nor analysed, as it fell outside of the scope of this study.

Effective DVRS could assist in early identification and response to IPV and may help alter the trajectory of increasing morbidity and mortality. Offering timely support and safety planning [41, 42] may also have the potential to reduce future presentations to ED [43]. Of the women in this cohort who had ED presentations in the 12-months prior to disclosing IPV, 30% had IVP documented at their ED presentation. Considering the morbidity and mortality associated with IPV, any opportunity to offer intervention and support within the health system, especially where multiple and complex needs are present, should be taken, provided the location for screening is safe [18, 25].

World Health Organization guidelines [18] on responding to IPV stipulate that it is essential that appropriate referral pathways be offered for women who disclosed through universal screening. In our study, all the women who disclosed IPV were offered a referral to social work and a discrete wallet-sized information card available in multiple languages. Of those women, 42% of those who disclosed IPV accepted the offer of a social work referral, and many accepted further referrals, including for accommodation and legal support. The women who did not require a support referral often identified to the clinician that they already had support or have previously had IPV support. The availability of a permanent social worker at the pilot site made DVRS feasible and, importantly, trauma informed. Currently, in Australia, only two public oral health services have an onsite social worker. This paucity of on-site social workers (or similar providers) creates a significant barrier to implementing DVRS across all oral health services and private clinics. Availability of on-site support or solid partnerships with local IPV services that could provide timely response would need to be considered for DVRS to expand to other oral health services or be implemented in private clinics. In NSW for public health services to screen these processes need to be approved by PARVAN, NSW Health and the local health district [18, 25, 26].

The average percentage of eligible women who received DVRS screening was low (5%) within both SDH clinics compared to other areas in NSW where screening is mandatory. For example, the last reported data for the same local health district mandatory screening areas varied between 98% of eligible women being screened in Maternity and 54% in Child and Family services [43]. During periods where the SDH clinics had competing priorities, such as accreditation or school holidays, we noted a decrease in the screening rate. A 2023 survey of SDH clinicians included in this study identified other barriers to DVRS implementation, including competing priorities, limited appointment times, no allocated Key Performance Indicators (KPIs), lack of privacy, clinician discomfort in asking questions and forgetting to screen if not prompted by the electronic system. Similar barriers were identified in a 2022 Australian feasibility study of DVRS in an emergency department [35]. These barriers would also need to be addressed to ensure success of DVRS implementation across the public oral health setting. The introduction of training, KPIs, incorporation of DVRS in the electronic health record and increased funding would go some way towards addressing these barriers.

### Limitations

Demographic and risk factor data collected was collated via the available medical and oral health records. Therefore, data available was based on health interventions and documentation described in these records. Where data was not available the results were listed as unknown. At SDH, the screening questions related to why the screen was not completed were inconsistently filled out by clinicians, so often the reason for not screening was unknown. In the Paediatric Dentistry Department, screens and forms were not completed if a male parent or carer attended the appointment. Therefore, the screening rate could be higher than the 5% indicated in the results, but this information was unable to be extracted.

Despite the participating sites having a large proportion of patients with a preferred language other than English, most screens were completed women who spoke English [44]. Other studies of IPV screening have highlighted that language is often a barrier to completing the screen [45]. Clinicians in our study identified that inability to have a face-to-face interpreter impacted on their capacity to screen non-English speaking women.

Despite these limitations, the study provides evidence to support the need for DVRS in public dental settings as these healthcare facilities may create opportunities for women to be offered support.

Due to the nature of IPV there is limited capacity to follow-up on outcomes of interventions and support offered to those who are screened. No time limitations were placed on women to receive support, and some made contact again months after their initial referral either directly to the social worker or other hospital staff, when new support needs arose.

## Conclusion

This study demonstrates that DVRS in public oral health services is an acceptable and effective way to identify women subjected to IPV and offer support. It has also highlighted the complex needs of women accessing oral health services who are subjected to IPV, and the similarities in risk between women accessing oral health and other NSW Health mandatory targeted areas. As implementation of DVRS requires the involvement of a suitably trained and qualified social worker, or equivalent provider of trauma informed support, placement of social workers (or similar) in oral health services implementing DVRS would need to be considered.

## Data Availability

The data supporting the findings of this study are not publicly available due to privacy and confidentiality considerations. Requests for access to the data should be directed to the corresponding author and will be considered on a case-by-case basis.
